# Flash‐Induced Stretchable Cu Conductor via Multiscale‐Interfacial Couplings

**DOI:** 10.1002/advs.201801146

**Published:** 2018-10-04

**Authors:** Jung Hwan Park, Jeongmin Seo, Cheolgyu Kim, Daniel J. Joe, Han Eol Lee, Tae Hong Im, Jae Young Seok, Chang Kyu Jeong, Boo Soo Ma, Hyung Kun Park, Taek‐Soo Kim, Keon Jae Lee

**Affiliations:** ^1^ Department of Materials Science and Engineering Korea Advanced Institute of Science and Technology (KAIST) 291 Daehak‐ro, Yuseong‐gu Daejeon 34141 Republic of Korea; ^2^ Department of Mechanical Engineering Korea Advanced Institute of Science and Technology (KAIST) 291 Daehak‐ro, Yuseong‐gu Daejeon 34141 Republic of Korea; ^3^ Division of Advanced Materials Engineering Chonbuk National University Jeonju Jeonbuk 54896 Republic of Korea; ^4^ Department of Industrial Design Korea Advanced Institute of Science and Technology (KAIST) 291 Daehak‐ro, Yuseong‐gu Daejeon 34141 Republic of Korea

**Keywords:** flash–material interactions, interlocking, stretchable conductors, wireless communication, wrinkling

## Abstract

Herein, a novel stretchable Cu conductor with excellent conductivity and stretchability is reported via the flash‐induced multiscale tuning of Cu and an elastomer interface. Microscale randomly wrinkled Cu (amplitude of ≈5 µm and wavelength of ≈45 µm) is formed on a polymer substrate through a single pulse of a millisecond flash light, enabling the elongation of Cu to exceed 20% regardless of the stretching direction. The nanoscale interlocked interface between the Cu nanoparticles (NPs) and the elastomer increases the adhesion force of Cu, which contributes to a significant improvement of the Cu stability and stretchability under harsh yielding stress. Simultaneously, the flash‐induced photoreduction of CuO NPs and subsequent Cu NP welding lead to outstanding conductivity (≈37 kS cm^−1^) of the buckled elastic electrode. The 3D structure of randomly wrinkled Cu is modeled by finite element analysis simulations to show that the flash‐activated stretchable Cu conductors can endure strain over 20% in all directions. Finally, the wrinkled Cu is utilized for wireless near‐field communication on the skin of human wrist.

Stretchable electronics have significant potential to shift personal computing toward new wearable and healthcare applications, such as robotic skin, conformal photovoltaics, and wireless communication systems.^1^, ^2^, ^3^, ^4^, ^5^, ^6^, ^7^ For instance, they can be mounted on curved and dynamic surfaces of the skin to monitor the body's vital signs through real‐time/bilateral data transmissions based on a human–machine interface (HMI), thus realizing a hyperconnected society.^8^, ^9^, ^10^, ^11^, ^12^, ^13^ Commercial wearable devices exist predominantly in the form of patches, bands, and types of textiles that yield strains associated with motions and detaching stresses in the wrist, elbow, and waist, which requires stretchability over 10–20%.^13^, ^14^, ^15^, ^16^, ^17^

Stretchable electrodes have been extensively developed using nanomaterials (e.g., metal nanowires (NWs), nanoparticles (NPs), carbon nanotubes (CNTs), and 2D materials), and geometrical engineering processes (e.g., buckled, serpentine, and net formations).^1^, ^2^, ^3^, ^8^, ^10^, ^12^, ^18^, ^19^, ^20^, ^21^, ^22^, ^23^, ^24^, ^25^, ^26^ However, structurally designed conductors face challenges with regard to wider applications due to their complicated encapsulation and transfer process, large interconnection area, and limited stretching directions.^27^, ^28^ Facile nanomaterial electrodes also suffer from their low conductivity (≈5.3 S cm^−1^), mechanical hysteresis, limited patternability, and poor dispersion uniformity.^24^, ^27^

Light–material interactions for flexible applications (e.g., inorganic‐based laser liftoff, photoreduction, light sintering, and low‐temperature polycrystalline silicon (LTPS)) have attracted considerable attention as a powerful solution for conducting electrodes on plastics.^29^, ^30^, ^31^, ^32^, ^33^, ^34^ They provide an outstanding capability to excite nonequilibrium and multiscale photon reactions within confined space and time for minimal damage on polymers, which can selectively tune the material morphology, chemical state, and interface configuration.^35^, ^36^, ^37^, ^38^ Several research groups have reported the monochromatic laser structuring of flexible conductors (e.g., Ag or Cu),^33^, ^39^, ^40^, ^41^ and have demonstrated the plasmonic welding of NW junctions.^42^ However, the developed NW conductor exhibits poor conductivity, percolation disuniformity, and low interconnection compatibility caused by its high level of roughness.^27^ Furthermore, the laser process has shown the lack of mass productivity due to the laser spot beam size and spatial energy variations.^30^ As an alternative, our group proposed the facile fabrication of a Cu conducting network on polymers using a broad‐spectrum flash lamp (from ultraviolet (UV) to infrared), offering the excellent advantages of an inexpensive light source with high output efficiency and large‐scale processability, including roll production.^34^ Nevertheless, Cu can be easily oxidized upon the photothermal interactions in air due to its low oxidation potential, which reduces the huge merits of low‐cost Cu material (100 times cheaper than Ag). In addition, a new strategy for stretchable and multiscale light–material interaction has yet to be exploited despite the fact that such a process is essential to realize harmonious integration of a human–machine interface.

Herein, we report a novel route by which to create stretchable conductors via the flash‐induced multiscale interface interactions between Cu and a polymer. Microscale randomly wrinkled Cu was formed on an elastomer substrate by irradiation of single pulse of millisecond flash light to enable Cu stretchability exceeding 20% in all directions. Nanoscale interlocked structure between the Cu NPs and the polymer can contribute to the substantial enhancement of stable Cu elongation under harsh stretching fatigue by increasing the Cu interfacial adhesion force. Simultaneously, photoreduction of CuO NPs and subsequent welding of Cu NPs were optimized by tuning the flash energy density and pulse width, resulting in excellent conductivity (≈37 kS cm^−1^) of the buckled stretchable electrode based on low‐cost Cu material in spite of its oxidation vulnerability. To the best of our knowledge, this electrical conductivity of the flash‐induced elastic Cu is more than 5 times higher than that of previous nanomaterial‐based stretchable conductors.^18^, ^19^, ^21^, ^24^, ^43^, ^44^ 3D structure of randomly wrinkled Cu was precisely modeled by controlling a compressing length and wrinkle mode (eigenvalue) in biaxial perturbation buckling step via a finite element analysis (FEA) simulations, which showed that the buckled Cu could relax the strain over 20% regardless of the 2D stretching directions. To present the practical utility of the elastic Cu conductor, near‐field wireless communication (NFC) on a human wrist was demonstrated by successfully transmitting pedometer data to a smartphone.

**Figure** **1**a schematically illustrates the overall concept of the fabrication of the stretchable Cu conductor on a polymer via flash‐induced multiscale interactions. A CuO NP ink was spin‐coated onto UV‐treated polydimethylsiloxane (PDMS) to form a CuO NP thin film on an elastomeric substrate. During the photoreduction of CuO NPs and subsequent sintering of Cu NPs by irradiation with a xenon flash light, two types of interfacial interactions between Cu and the polymer could occur, as presented in Figure 1a‐i. Microscale wrinkled Cu could form on an elastomer through the flash‐induced heating and cooling interactions between Cu and the polymer interface, producing thermal compressive strain on the Cu/PDMS. Simultaneously, a nanoscale interlocked structure arose between the Cu and the elastomer due to the flash‐activated rapid melting/solidification of the Cu and the polymer interface. Hence, an elastic and reversible Cu electrode could be demonstrated on the PDMS, as shown in Figure 1a‐ii. Figure 1b presents the resistivity of the fabricated Cu on a polymer by the flash exposure. The specific resistance of the CuO film decreased remarkably from ∞ to 27.2 µΩ cm through the sufficient CuO NP photoreduction and sequential Cu NP welding via flash light irradiation (energy density of 31.7 J cm^−2^ and pulse width of 25 ms). Figure 1c shows the X‐ray diffraction (XRD) measurement results of the CuO NP thin films (with a reducing agent, ethylene glycol), that was treated by a flash light (pulse width of 25 ms) with different energy densities of 0, 27.2, 29.6, and 31.7 J cm^−2^. As the light energy density increased from 0 to 31.7 J cm^−2^, the CuO NPs on PDMS were significantly reduced to Cu NPs within milliseconds by the flash‐induced successive dehydration reaction between the CuO NPs and the reducing agent.^34^ The energy dispersive X‐ray spectroscopy (EDS) mapping and spectrum results of the flash‐activated Cu present that the oxygen and carbon (organic) elements which existed throughout the CuO NP ink could be extracted and decomposed, respectively, by the photothermal interaction (Figure 1d and the insets of Figure 1e). The morphology of the CuO NPs (top) and the flash‐induced Cu electrode (bottom) are shown in the plane‐view scanning electron microscopy (SEM) images presented in Figure 1e. Unlike pristine CuO NPs, the flash‐activated Cu shows a welded structure (necking and fusing) between neighboring Cu NPs, achieving outstanding Cu conductivity by expanding the current path of the Cu electrode. Laser sintering of Ag and Au nanomaterials can also demonstrate high‐conductivity conductors, however, the poor scalability of the laser process as well as the expensive price of noble metals inhibits the practical applications in macroelectronics.

**Figure 1:**
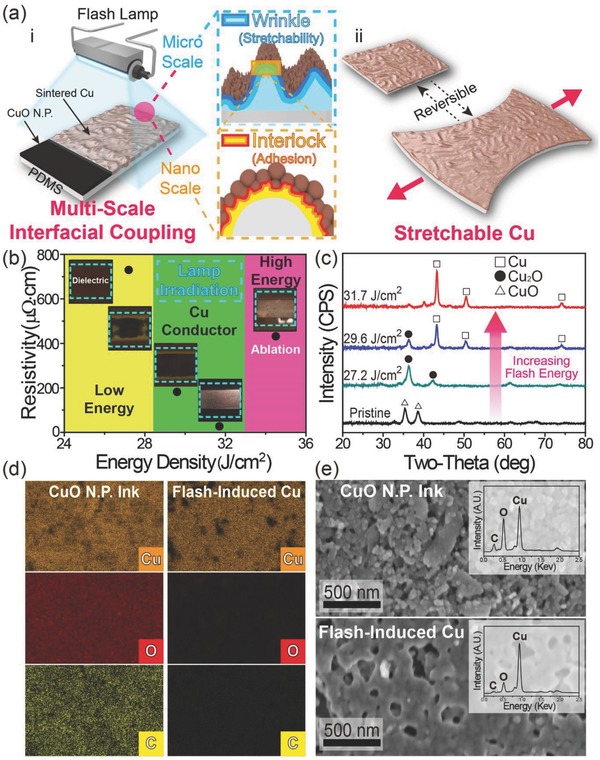
a) Schematic illustrations of the stretchable Cu conductor fabricated by flash‐induced multiscale modulation of the Cu and elastomer interface occurring during a photothermal reduction of CuO NPs and subsequent sintering of the Cu NPs. b) The resistivities of the elastic Cu electrodes on PDMS subjected to different flash light energy densities ranging from 24.8 to 34.3 J cm^−2^ (pulse width of 25 ms). The inset shows photographic images of the CuO NP films after irradiation with the corresponding flash light. c) XRD patterns of CuO NPs that were processed by flashes of light (pulse width of 25 ms) with different energy densities of 0, 27.2, 29.6, and 31.7 J cm^−2^. d) EDS mapping results of CuO NP ink and flash‐induced Cu (Cu: copper, O: oxygen, and C: carbon). e) Plane‐view SEM images of the CuO NPs (top) and the flash‐activated Cu (bottom) on polymer substrates. The insets show the EDS spectrums of the corresponding CuO NP ink (top) and the flash‐induced Cu conductor (bottom).

The thermal interaction induced by the flash light can also impose compressive stress on the Cu and the elastomer by rapidly heating and cooling the polymer interface, resulting in microscale randomly buckled Cu.^45^, ^46^ To investigate the stretchability of the Cu conductor with the random wrinkle formation, the 3D Cu structure on PDMS was modeled with a biaxial perturbation buckling step (**Figure** **2**a, right side) by modulating a compressing length and wrinkle mode (eigenvalue). The wavelength and amplitude of the wrinkled Cu model were determined as 44.14 and 5.61 µm, respectively, based on the photothermal simulation result and theoretical buckling mechanics (detail procedures are shown in the Experimental Section).^47^ As presented in Figure 2a, the buckled Cu could be more easily elongated and stretched than the Cu film without wrinkles. It underwent strain of less than 3–4% throughout the electrode at a yielding strain of 20%, which was lower than the fracture strain (≈5%) of Cu.^46^, ^48^ The porous Cu structure with a lower elastic modulus than bulk Cu has substantial potential to further release the strain during the tensile stress is applied. The elongation capability could be maintained regardless of the stretching direction due to the random wrinkle formation of the elastic Cu, as shown in the top of Figure 2b. The Figure 2b (bottom, left) presents the microscopic image of the experimentally demonstrated buckled Cu on elastomer by flash lamp processing. As shown in Figure S1 (Supporting Information), the wavelength and amplitude of the Cu were ≈45 and ≈5 µm, respectively, which agreed well with the theoretically estimated Cu model. The elastic Cu conductors could yield stably without degradation even when the 20% strain was applied along the horizontal, vertical, and diagonal directions, as presented in the bottom of Figure 2b. As shown in top‐view SEM images of Figure 2c, the flash‐induced photothermal interactions caused the 2D compressing stress to the interface between Cu and elastomer, resulting in a randomly wrinkled Cu structure without crack formation. Figure 2d presents cross‐sectional focused‐ion beam SEM (FIB‐SEM) images of the unstretched (top) and stretched (bottom) wavy Cu. As illustrated by the SEM image at the bottom of Figure 2d, the flash‐induced wrinkled Cu could be elongated without crack and delamination of the Cu conductor despite yielding strain of 20%. As described in Figure 2e, the Cu electrode could be conformally fabricated onto the PDMS substrate in spite of its highly curved surface, as formed during the flash‐induced polymer wrinkling process. Furthermore, the magnified SEM image of the cross‐section of the elastic Cu in Figure 2f shows that the nanoscale interlocked formation could be caused by the flash‐activated instantaneous melting and solidification of the Cu and polymer interface. The rate of PDMS interaction with oxygen was significantly reduced because the flash lamp processing is too rapid to provide optimal kinetics and sufficient oxygen dissolution reaction for the thermal oxidative degradation of PDMS polymer.^49^ The flash‐induced interfacial interlocking in nanoscale between Cu and the elastomer enabled to achieve higher Cu adhesion properties than that of vacuum‐deposited Cu conductor on PDMS, as confirmed by a simple tape test (Figure S2, Supporting Information). This good adhesion of the interlocked Cu can be attributed to the large bonded area between the Cu nanostructures and the polymer surface,^34^ which is important not only for stable adhesion of the Cu on an elastomer substrate but also for high stretching durability of the Cu by preventing the Cu from delaminating due to elastic deformation. The stretching durability of the buckled Cu was evaluated by monitoring the normalized resistance of the stretchable Cu during the application of external strain from 0% to 40%, as presented in Figure 2g. The normalized resistance of the sputtered Cu on PDMS without wrinkles increased critically to ≈26.6 within 5% strain and reached ≈33 000 at 7.5% strain. On the other hand, the normalized resistance of the wrinkled elastic Cu exhibited little change even at 20% strain. The conductivities at the maximum endurable strain for state‐of‐the‐art nanomaterial‐based stretchable electrodes are compared in Figure 2h.^18^, ^19^, ^21^, ^24^, ^43^, ^44^ As far as we know, the flash‐induced multiscale Cu conductor exhibits more than 5 times higher conductivity (≈37 kS cm^−1^) than that of previously reported stretchable nanomaterial electrodes, fulfilling the stretchability requirement (20%) for commercial wearable electronics. This outstanding conductivity of elastic Cu beyond the intrinsic limitations (e.g., yield strength, Young's modulus, and Poisson's ratio) was ascribed by multiscale photothermal interaction that controls physical mechanics of materials. We believe that the elastic capability of the flash‐activated stretchable Cu can be further improved by tuning the wrinkle structure (wavelength and amplitude), modulus of the substrate, and film thickness. The reversibility and stability of the stretchable Cu under yielding stress was examined by applying repeated stretching (20% strain) and unstretching fatigue (Figure S3, Supporting Information). While the normalized resistance of vacuum‐deposited Cu on an elastomer was irreversibly increased to ≈650 after 10 stretching cycles, the stretchable Cu conductor created via flash light interaction presents high reliability without mechanical hysteresis despite 100 cycles of yielding stress.

**Figure 2:**
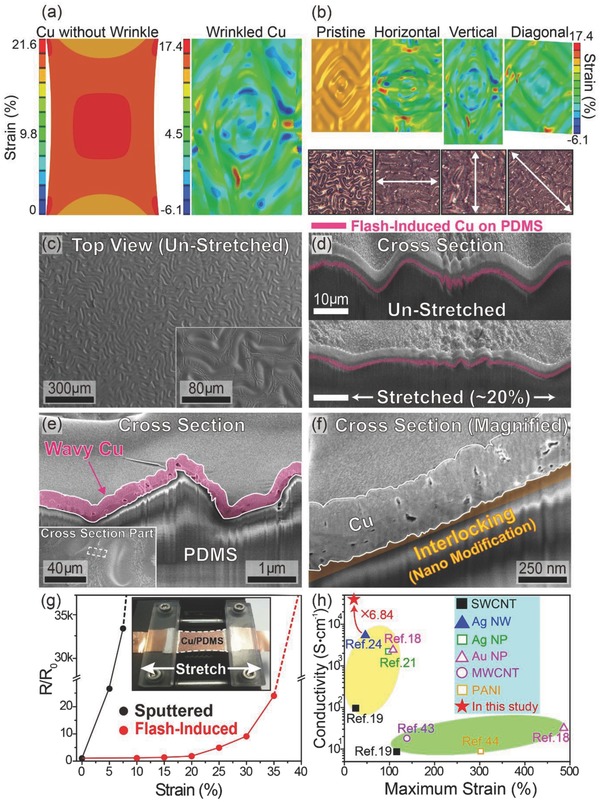
a) The strain distribution results (FEA simulations) after stretching (vertical stretching direction, 20% applied strain) for the Cu without wrinkles (left side) and the flash‐induced Cu conductor (right side). b) Top side: the FEM modeling result of the buckled Cu (left), and the FEM simulation results after inducing 20% strain in horizontal, vertical, and diagonal directions to the wrinkled Cu model (right). Bottom side: the microscopic image of the wrinkled Cu (left, after applying 0% strain), and the microscopic images of the buckled Cu after applying 20% strain in horizontal, vertical, and diagonal directions (right). c) Top‐view SEM image of wrinkled Cu on an elastomer substrate created by the flash lamp process. The inset shows a magnified SEM image of a buckled Cu electrode. d) SEM images showing the cross‐section of unstretched (top) and stretched wrinkled Cu conductors. e) Magnified cross‐sectional SEM image of flash‐induced wavy Cu on a polymer substrate. The inset shows a SEM image of a tilted view of the buckled Cu. f) SEM image of the magnified cross‐section of a flash‐activated interlocked structure arising between Cu and an elastomer interface. g) The normalized resistance of sputtered and flash‐induced Cu during yielding strain up to 40% was applied. The inset shows an optical image of a stretchable Cu electrode in a stretched state. h) A comparison work to recent studies in elastic conductors. Data points were excerpted from the following papers: black filled square,^19^ blue filled triangle,^24^ green open square,^21^ magenta open triangle,^18^ purple open circle,^43^ orange open square,^44^ red filled asterisk (this study).

To study the Cu adhesion enhancement effect by the interlocked Cu on polymer substrates, a Cu electrode was fabricated on a polyimide (PI) substrate using the flash lamp, and this was followed by quantitative measurements of the Cu delamination fracture force using the double‐cantilever‐beam (DCB) method.^29^ Note that PDMS substrate could not be utilized to semiquantitatively monitor the pure adhesion enhancement phenomena induced via interlocked interface between Cu and polymer due to the excessive plastic deformation of the elastomer. In addition, the wrinkled structure of the flash‐induced Cu on PDMS inhibits the investigation of the Cu nano‐interlocking effect on various mechanical properties such as adhesion, and reliabilities under bending or environmental fatigues. **Figure** **3**a shows cross‐sectional SEM images of CuO NP ink (top) and a flash‐induced Cu electrode (bottom) on a PI film. As shown in the SEM image at the bottom of Figure 3a, an interlocking structure was also induced between the Cu and polymer interface during the photothermal reduction of CuO NPs and the sequential sintering of Cu NPs. This flash‐activated Cu was used to prepare DCB peel test specimens by slicing it into pieces 10 mm in width and 30 mm in length. As illustrated in the inset of Figure 3b, Al beams (thickness of 3 mm) were bonded to the side of a plastic substrate and a Cu electrode using an epoxy adhesive. The ends of Al beams were pulled by a linear actuator at a constant displacement rate of 0.1 mm s^−1^ to measure the peeling force of the Cu in J m^−2^ (inset of Figure 3b). The measured adhesion energy of the Cu on a polymer substrate was 289.4 J m^−2^, exhibiting greater adhesion force by 201% than that of sputtered Cu on PI (Figure 3b). Figure 3c presents the electrical properties of the flash‐induced Cu on a polymer film, which shows high durability of the Cu electrode under bending fatigue (10 000 bending cycles at a bending radius of 5 mm) compared to vacuum‐deposited Cu on a plastic substrate. As described in Figure 3d‐iii, severe cracks were created on the sputtered Cu after bending stress. On the other hand, no fracture damage was observed for the flash‐activated Cu conductor, stemming from the excellent adhesion properties of the dense Cu on the polymer (Figure 3d‐iv).^50^, ^51^ The significant enhancement of the Cu adhesion energy could also improve the life spans of electrodes^52^ in humid condition. Figure 3e shows the results of an accelerated humidity‐resistance test conducted with the theoretical Arrhenius approach to calculate the lifetime of flash‐induced Cu on a polymer (PI). The Cu electrode created via the flash light process was placed in an environment of 85% humidity at three different temperatures of 75, 85, and 95 °C. If humid moisture (85%) at a high temperature (from 75 to 95 °C) infiltrates into the samples through the vulnerable Cu and PI interface, the Cu metal line would be destroyed by corrosion, which would then induce delamination, breakage, and oxidation of the Cu. The time‐to‐failure (*t*_f_) was defined as the length of time until the resistance became 10% higher than the initial status after putting the fabricated Cu into the humid chamber.^52^ The *t*_f_ of the flash‐induced Cu conductors in humid chamber (85%) at different temperatures of 75, 85, and 95 °C were 1, 3, and 9 h, respectively. Figure 3e shows an Arrhenius curve based on the linear fitting of −ln(*t*_f_) versus 1000/*T* to estimate the failure time of the flash‐activated Cu at 25 °C. An Arrhenius equation and the accelerated factor (AF) between two temperatures are given as Equations (1) and (2), 53

(1) $$\ln\left(\frac{1}{t_{f}}\right)\;=\;\ln\left(A\right)\;-\;\frac{E_{a}}{kT}$$

(2) $$\mathrm{AF}\;=\;e^{\left(\frac{E_{a}}{k}\right)\left(\frac{1}{T_{2}}\;-\;\frac{1}{T_{1}}\right)}\;=\;\frac{t_{f2}}{t_{f1}}$$

where *A*, *E*_a_, *k*, and *T* are the pre‐exponential factor, the activation energy (eV) for the failure of flash‐induced Cu, Boltzmann's constant, and the temperature in degrees Kelvin, respectively. The activation energy was calculated and found to be 1.24 eV from the slope (*E*_a_·*k* ^−1^) of the Arrhenius graph in Figure 3e, which is mainly determined by the Cu interfacial bonding strength on the polymer film and the water absorption rate at the Cu and plastic interface. The *t*_f_ value of the flash‐activated Cu electrode at room temperature (25 °C) can be predicted by Equation (4) by substituting into the equation two selected temperatures of 95 and 25 °C, and *t*_f_ of the flash‐induced Cu conductor at 85% and 95 °C. From the accelerated humidity‐resistance test and the Arrhenius model, the life span of the Cu on a polymer substrate by flash exposure was estimated to be 10 280 h at 25 °C, presenting 60 times greater material stability than that of the vacuum‐deposited Cu on PI in humid environment. The X‐ray photoelectron spectroscopy (XPS) depth profile result of the sputtered Cu in Figure S4a (Supporting Information) shows that severe oxidation damage occurs not only on the Cu surface but also at the Cu and polymer interface. This indicates that the adhesion of weakly bonded Cu onto the PI could be easily degraded by the environmental stress (e.g., polymer dehydration), resulting in Cu delamination from PI and therefore creating additional oxidation and crack sites.^52^, ^54^ As illustrated in Figure 3f‐iii, the sputtered Cu with low adhesion on a polymer could easily peel due to environmental fatigue (85% humidity at 85 °C for 96 h), allowing the permeation of moisture through the Cu interface and thus causing islands to form of the Cu material. In contrast, the highly adhesive Cu electrode created by the flash light method showed only minor amounts of crack damage and interfacial Cu oxidation (Figure 3f‐iv and Figure S4b (Supporting Information)). Note that this humidity resistance can be further improved by using fluorinated polymer or chloroprene substrates that are not permeable to humid environment.

**Figure 3:**
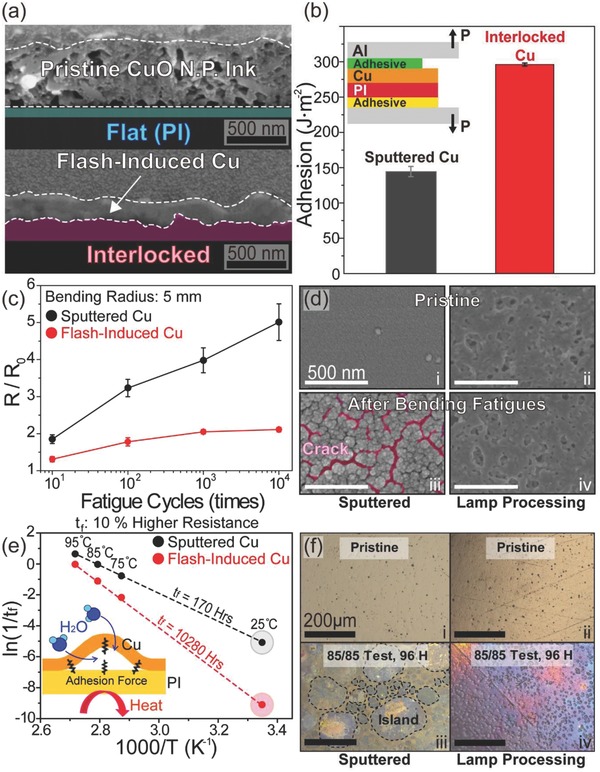
a) SEM images showing the cross‐section of the CuO ink (top) and flash‐activated Cu (bottom) on polymer substrates. b) The adhesion energy measurement results of the sputtered and the flash‐induced Cu as evaluated by the DCB method. The inset shows structure of the DCB specimen and the loading direction for the DCB peel test. c) The normalized resistance of the sputtered and flash‐activated Cu on polymer substrates during a cyclic bending test (bending radius: 5 mm). d) SEM images of the sputtered and flash‐induced Cu before and after 10 000 cycles of bending fatigue. e) Arrhenius plot of ln(1/*t*_f_) versus 1000/*T*, presenting the temperature‐dependent stability of the sputtered and the flash‐activated Cu on polymer substrates. f) Microscopic images of sputtered and flash‐induced Cu before and after an accelerated humidity‐resistance test (85% humidity at 85 °C for 96 h).

To verify the practical usefulness of the flash‐induced elastic electrodes, we demonstrated a stretchable Cu antenna on skin of human wrist, as shown in **Figure** **4**a. First, the coated CuO NP film on PDMS was shaded by a metal mask with a spiral antenna pattern to irradiate a flash of light selectively onto CuO NPs. The geometric parameters of the antenna (e.g., number of turns, and mean coil diameter) for a NFC pedometer system were custom‐designed based on the electrical properties of wrinkled elastic Cu. Second, the stretchable Cu antenna was resulted by rinsing CuO ink (where the flash light is not exposed) off. Finally, the Cu antenna for wireless data transmission was interconnected to the aforementioned pedometer system. To estimate the resonance center frequency (RF, *f*_c_) and quality factor (*Q*‐factor,$\;\frac{f_{c}}{\Delta f}$) of the antenna under the stretching deformation, tensile stress was applied while the reflection coefficient *S*_11_ was ascertained by a network analyzer (Figure 4b). As presented in Figure 4c, the *f*_c_ and *Q*‐factor values of the antenna with the RF matching circuit were 13.4 MHz and 14.6 at 0% strain, respectively. The elastic antenna exhibited stable NFC properties (*f*_c_: from 13.4 to 12.6, and *Q*‐factor: from 14.6 to 10.57) regardless of the elongation strain from 0% to 20%. This good reliability of the stretchable Cu antenna sufficiently satisfied the theoretical requirements for successful wireless transmission (12.2 < *f*_c_ < 14.92, 10 < *Q*‐factor < 20).^55^ It is essential that the antenna survives for hundreds of stretching cycles to realize a robust wearable communication device. As shown in Figure 4d, there were no marked differences in the antenna *f*_c_ and *Q*‐factor values during 1000 cycles of 20% yielding strain. Figure 4e illustrates the NFC pedometer system including the antenna, RF matching circuit, microcontroller unit (MCU), and sensor modules. Despite severe stress caused by the wrist joint, the elastic Cu antenna successfully transmitted the pedometer data (the number of steps over time, Figure 4g) to a smartphone, as presented in Figure 4f and Movie S1 (Supporting Information), which proves that the elastic Cu created by the flash lamp process can be practically integrated into electronic‐skin (e‐skin) devices.

**Figure 4:**
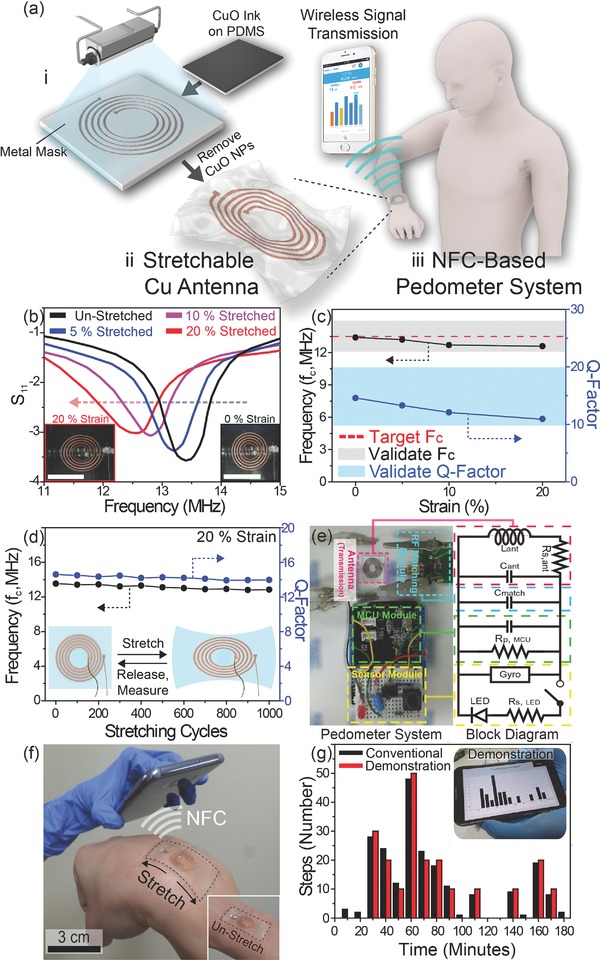
a) Schematic illustrations of the fabrication procedures of the NFC pedometer system based on the flash‐induced stretchable Cu antenna. b) The reflection coefficient of the elastic Cu antenna under tensile strain ranging from 0% to 20%. The inset shows photographic images of the stretchable Cu antenna before (bottom right) and after (bottom left) the application of 20% yielding strain. Scale bars of the inset photos are 1.5 cm. c) The *f*_c_ and *Q*‐factor values of the antenna under stretching deformation (elongation strain from 0% to 20%). d) The *f*_c_ and *Q*‐factor values of the stretchable antenna during 1000 cycles of 20% stretching fatigue. The inset (schematics) shows the reliability evaluation procedures for the elastic Cu antenna on an elastomer substrate under repeated 20% yielding strain. e) A photographic image of the NFC pedometer system (left side), and corresponding block diagram (right side). f) An image of the stretched Cu antenna when bent at the wrist during NFC wireless signal transmission of pedometer data to a smartphone. The inset shows a photo of the unstretched Cu antenna on the wrist. g) The pedometer data (number of steps for 3 h) acquired during the demonstration of our NFC system and a conventional pedometer device. The inset photo shows the pedometer data received through NFC by the stretchable Cu antenna.

In summary, we demonstrated a flash‐induced stretchable and multiscale Cu conductor with excellent conductivity (≈37 kS cm^−1^) and good elongation capabilities (≈20%). A flash light with an energy density level of 31.7 J cm^−2^ and a pulse duration of 25 ms facilitated the optimized photoreduction of CuO NPs, followed by the densification of the Cu NPs, resulting in a Cu conductor with outstanding resistivity on an elastomeric substrate. A microscale randomly buckled Cu structure (wavelength of ≈45 µm and amplitude of ≈5 µm) was formed on PDMS through thermal compressive strain induced by a millisecond flash light, which realizes Cu stretchability exceeding 20% in all directions. Simultaneously, a nanoscale interlocked interface between the Cu NPs and a polymer strengthened the Cu adhesion on the elastomer, preventing Cu delamination due to elongation fatigue. The effects of Cu nano‐interlocking on a polymer substrate (e.g., lifetime and reliability) could be investigated by evaluating the interfacial fracture force, resistance to humidity, and bending durability of flash‐activated Cu on a polymer (PI). In addition, 3D FEA simulations indicated that the wrinkled Cu conductor could absorb yielding strain over 20% regardless of the stretching direction. The buckled Cu created via the flash lamp process was used for near‐field wireless communications (13.56 MHz) on the skin of the wrist of a human volunteer, stably transmitting pedometer data to a smartphone under harsh strain imposed when the volunteer bent his wrist joint. This novel flash‐induced stretchable technology can provide an important tool for elastic electronics, such as wrinkled e‐skin and healthcare sensors.

## Experimental Section

*Photothermal Simulation*: The heat flux simulation was conducted using COMSOL Multiphysics 5.3 software. During pulsed flash irradiation, the temperature distribution in the elastomer interface was determined by the following Equation (3)

(3) $$Q\;=\;\rho C\frac{\partial T}{\partial t}\;+\;\rho C\cdot \nabla T\;-\;\nabla \cdot \left(k\nabla T\right)$$

where *Q* is the total heat generated by the flash light and ρ, *k*, and *C* are the density, thermal conductivity, and thermal capacity of the materials (CuO, PDMS, and air), respectively. The entire configuration of the CuO NPs (Novacentrix) on the polymer substrate was modeled considering the size and shape of the CuO NPs (spherical, 100 nm diameter), the number of CuO NP layers, and the thickness of the PDMS substrate. The density, thermal conductivity, and thermal capacity at constant pressure of CuO NPs was 6310 kg m^−3^, 32.9 W m^−1^ K^−1^, and 550.5 J kg^−1^ K^−1^, respectively.^56^ The experimentally applied flash light ignition condition (function of rectangular light pulse, pulse width of 25 ms, and energy density of 31.7 J cm^−2^) was used to simulate the photothermal effects within the Cu and elastomer interface. Prior to the heat flux simulation of solid module, the entire system was configured and maintained at the constant atmosphere pressure of 1 atm and the initial temperature of 293.15 K.

*Theoretical Buckling Mechanics*: Figure S5 (Supporting Information) shows the FEA simulation result to investigate the flash‐induced heat distribution at the polymer interface. Intense heat (average temperature of 773 K) could diffuse into the elastomer (up to 670 µm in depth), extending the PDMS by thermal expansion. After the pulsed flash irradiation was completed, the expanded Cu/polymer was subsequently cooled and contracted, which produced thermal compressive stress on the Cu and elastomer. The thermal strain imposed on the Cu on polymer substrate by flash lamp processing was calculated to be ≈14% according to Equation (4), 45

(4) $$\Delta \alpha \Delta T\;=\;\left(\alpha_{\mathrm{PDMS}}\;-\;\alpha_{\mathrm{Cu}}\right)\;\times \;\Delta T$$

where α_PDMS_ and α_Cu_ are correspondingly the coefficients of thermal expansion of the PDMS substrate and the Cu film, and Δ*T* represents the increase in the temperature of the polymer. The surface temperature profile of the PDMS could be controlled by flash lamp processing parameters (e.g., energy density and pulse width). For relatively large amounts of wrinkling strain (>10%), the wavelength of the buckled Cu could be theoretically estimated using Equation (5), 43, 57

(5) $$\lambda \;=\;\frac{\lambda_{0}}{\left[\left(1\;+\;\varepsilon_{\mathrm{pre}}\right){\left(1\;+\;\xi \right)}^{\frac{1}{3}}\right]}$$

Here, λ_0_ =$2\pi h_{f}{\left[\frac{{\bar{E}}_{f}}{3{\bar{E}}_{s}}\right]}^{\frac{1}{3}}$, $\xi \;=\;\frac{5}{32}\;\varepsilon_{\mathrm{pre}}$ (1 + ε_pre_), ε_pre_ denotes the thermal prestrain, *h*_f_ is the thickness of the Cu film, and ${\bar{E}}_{f}$ and ${\bar{E}}_{s}$ are correspondingly the plain‐strain moduli of Cu and PDMS. The amplitude of the stretchable Cu, *A* =$\frac{A_{0}}{\sqrt{1\;+\;\varepsilon_{\mathrm{pre}}}{\left(1\;+\;\xi \right)}^{\frac{1}{3}}}\;\;\left(\mathrm{where}\;\;A_{0}\;=\;h_{f}\;\sqrt{\frac{\varepsilon_{\mathrm{pre}}}{\varepsilon_{c}}\;-\;1}\right)$, depends on the film thickness, prestrain, and the critical buckling strain $\varepsilon_{c}\;=\;\frac{1}{4}{\left(\frac{3{\bar{E}}_{s}}{{\bar{E}}_{f}}\right)}^{2/3}$.

*Pedometer System*: A wireless communication board (Arduino, NFC shield V 2.0) was purchased for NFC at 13.56 MHz. An inclination sensor (Hedong, SW‐200) was also bought and integrated onto the NFC board to detect stepping motion by the user. A flash‐induced antenna with inductance of 450 nH was connected in parallel to a RF matching circuit (capacitance: 220 pF) and a NFC board (capacitance of MCU and printed circuit board: 86 pF), resulting in an antenna reflection coefficient spectrum with a center frequency of 13.4 MHz. After completing the hardware system, Android Arduino programming was done to save the pedometer data of the inclination sensor in the MCU and they were sent to a smartphone via NFC. An APK software file for Android applications was developed and installed on the smartphone to receive the data of the pedometer system. The authors would like to acknowledge Dr. D. J. Joe for his spontaneous support with photography and video work. Informed consent was obtained.

*Characterizations*: The morphologies of the stretchable Cu conductors were observed by means of SEM (Magellan 400, FEI) and FIB‐SEM (Helios Nanolab 450 F1, FEI, Kaist Analysis Center for Research Advancement (KARA)). The chemical and elemental compositions of the CuO NP ink and the flash‐induced Cu electrodes were monitored with XRD (K‐Alpha, Thermo Fisher Scientific), EDS (Magellan 400, FEI), and XPS (K‐Alpha, Thermo VG Scientific). The inductance, capacitance, and reflection coefficient of the antennas were measured by a multifrequency LCR meter (Hewlett Packard, Agilent 4275A) and a network analyzer (Hewlett Packard, Agilent 8753E). Electrical characterizations were performed by using a Keithley 4200‐SCS device.

## Conflict of Interest

The authors declare no conflict of interest.

## Supporting information

Supplementary

Supplementary

Supplementary
