# Supplementary material for: Flash‐Induced Stretchable Cu Conductor via Multiscale‐Interfacial Couplings
Source: Adv Sci (Weinh). 2018 Oct 4;5(11):1801146. doi: 10.1002/advs.201801146 (PMC6247032; doi:10.1002/advs.201801146)
Supplement: Supplementary file 1 — Supplementary [file ADVS-5-1801146-s002.pdf]

Copyright WILEY-VCH Verlag GmbH & Co. KGaA, 69469 Weinheim, Germany, 2018.

ADVANCED SCIENCE Open Access

Supporting Information

for *Adv. Sci.*, DOI: 10.1002/advs.201801146

Flash-Induced Stretchable Cu Conductor via Multiscale-Interfacial Couplings

*Jung Hwan Park, Jeongmin Seo, Cheolgyu Kim, Daniel J. Joe, Han Eol Lee, Tae Hong Im, Jae Young Seok, Chang Kyu Jeong, Boo Soo Ma, Hyung Kun Park, Taek-Soo Kim, and Keon Jae Lee**

Copyright WILEY-VCH Verlag GmbH & Co. KGaA, 69469 Weinheim, Germany, 2016.

Supporting Information

Flash-Induced Stretchable Cu Conductor via Multiscale-Interfacial Couplings

*Jung Hwan Park, Jeongmin Seo, Cheolgyu Kim, Daniel J. Joe, Han Eol Lee, Tae Hong Im, Jae Young Seok, Chang Kyu Jeong, Boo Soo Ma, Hyung Kun Park, Taek-Soo Kim, Keon Jae Lee**

Dr. J. H. Park, Dr. D. J. Joe, H. E. Lee, T. H. Im, Prof. K. J. Lee
Department of Materials Science and Engineering, Korea Advanced Institute of Science and Technology (KAIST), 291 Daehak-ro, Yuseong-gu, Daejeon, 34141, Republic of Korea
E-mail: keonlee@kaist.ac.kr

Dr. J. Seo, Dr. C. Kim, J. Y. Seok, B. S. Ma, Prof. T.-S. Kim
Department of Mechanical Engineering, Korea Advanced Institute of Science and Technology (KAIST), 291 Daehak-ro, Yuseong-gu, Daejeon, 34141, Republic of Korea

Prof. C. K. Jeong
Division of Advanced Materials Engineering, Chonbuk National University, Jeonju, Jeonbuk 54896, Republic of Korea

H. K. Park
Department of Industrial Design, Korea Advanced Institute of Science and Technology (KAIST), 291 Daehak-ro, Yuseong-gu, Daejeon, 34141, Republic of Korea

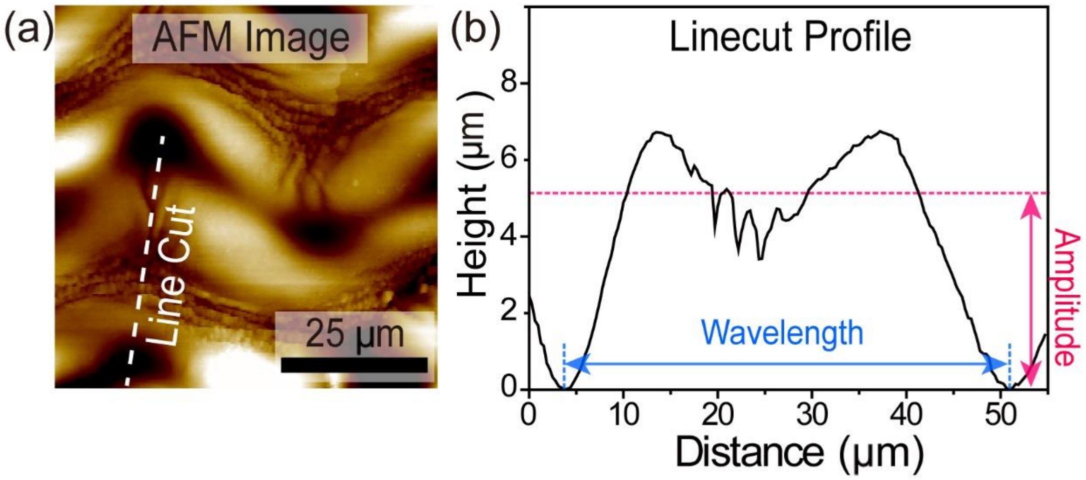

Figure S1. a) 2D AFM image of the buckled Cu via flash light irradiation. b) Line-cut AFM profile of the wrinkled elastic Cu electrode.

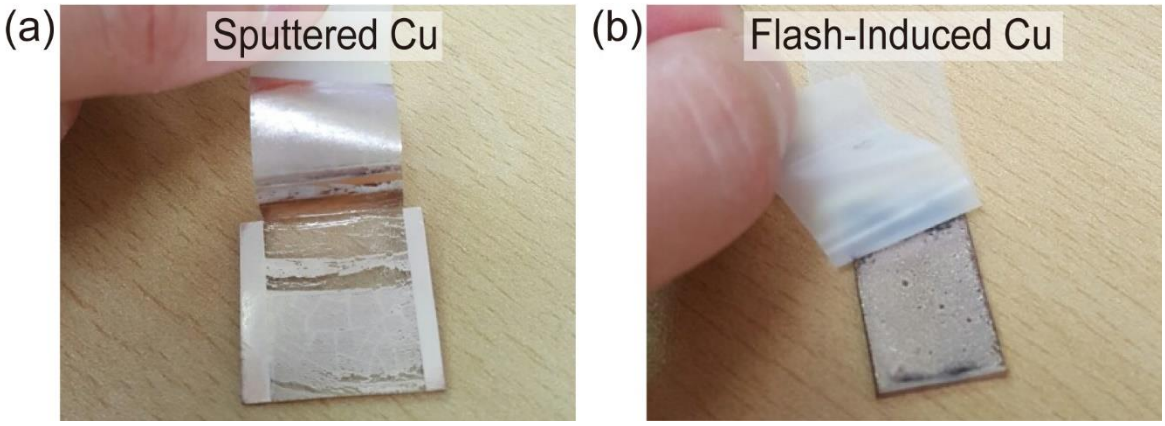

Figure S2. Tape peel test (3M tape) of the sputtered Cu and the flash-induced Cu on elastomer substrates. It shows that the nano-interlocked Cu by flash light irradiation has much higher adhesion than that of the sputtered Cu.

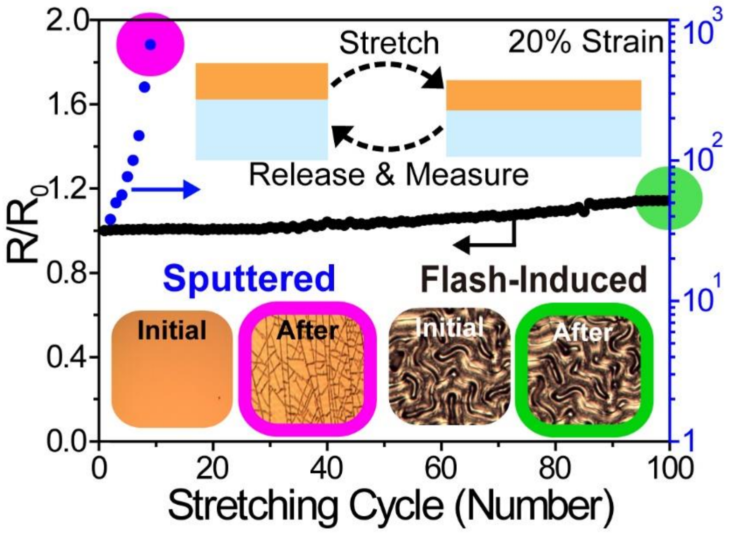

Figure S3. The normalized resistance of sputtered and flash-activated Cu on PDMS substrates during a cyclic stretching test (applied strain: 20 %). The schematics in the top inset of figure S3 present the procedures used for the examination of the reversibility of the Cu conductors on the elastomer under repeated 20 % yielding strain. The bottom inset shows microscopic images of the sputtered and flash-induced Cu before and after the stretching cycle test. The resistivity of the sputtered Cu on an elastomer (~2 μΩ·cm) was increased to ~1300 μΩ·cm after ten stretching cycles.

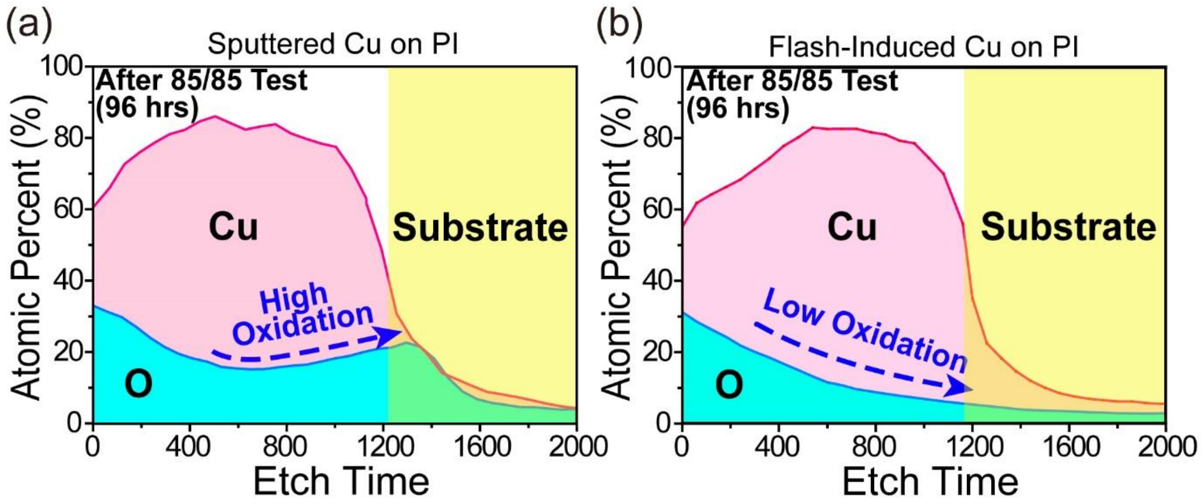

Figure S4. XPS depth profile results of (a) the sputtered Cu and (b) the flash-induced Cu on PI.

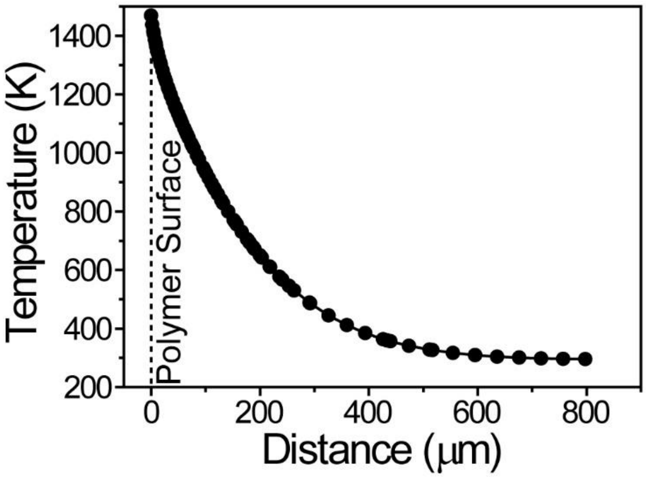

Figure S5. The flash-induced heat distribution in the polymer interface. The experimental light irradiation condition (energy density of 31.7 J·cm^{-2}, and pulse width of 25 ms) was used.
